# Understanding How University Students Use Perceptions of Consent, Wantedness, and Pleasure in Labeling Rape

**DOI:** 10.1007/s10508-020-01772-1

**Published:** 2020-07-08

**Authors:** Peter J. Hills, Megan Pleva, Elisabeth Seib, Terri Cole

**Affiliations:** 1 Department of Psychology, Bournemouth University, Talbot Campus, Fern Barrow, Poole, Dorset, Bournemouth, BH12 5BB UK; 2 Department of Psychology, Anglia Ruskin University, Cambridge, UK

**Keywords:** Consent, Rape, Wantedness, Pleasure, Sex scripts, Rape myths

## Abstract

While the lack of consent is the only determining factor in considering whether a situation is rape or not, there is sufficient evidence that participants conflate wantedness with consent and pleasurableness with wantedness. Understanding how people appraise sexual scenarios may form the basis to develop appropriate educational packages. We conducted two large-scale qualitative studies in two UK universities in which participants read vignettes describing sexual encounters that were consensual or not, wanted or unwanted and pleasurable or not pleasurable. Participants provided free-text responses as to whether they perceived the scenarios to be rape or not and why they made these judgments. The second study replicated the results of the first and included a condition where participants imagined themselves as either the subject or initiator of the sexual encounter. The results indicate that a significant portion of our participants held attitudes reflecting rape myths and tended to blame the victim. Participants used distancing language when imagining themselves in the initiator condition. Participants indicated that they felt there were degrees of how much a scenario reflected rape rather than it simply being a dichotomy (rape or not). Such results indicate a lack of understanding of consent and rape and highlight avenues of potential educational materials for schools, universities or jurors.

**Electronic supplementary material:**

The online version of this article (10.1007/s10508-020-01772-1) contains supplementary material, which is available to authorized users.

## Introduction

While the definition of rape is something that many people believe they understand across many countries, there is a significant amount of data that indicate misunderstandings of the practice.^1^ These misunderstandings may result from the differences between the stereotype of a rape and what typically occurs: for example, whereas the stereotypical rape is committed by a stranger (Grubb & Harrower, 2008; Hockett, Saucier, & Badke, 2015; Robinson, 2008), 90% of rape victims are aware of the identity of their attacker (Koss, Gidycz, & Wisniewski, 1987; Ministry of Justice, 2013; Muehlenhard & Linton, 1987; National Victim Center, 1992; O’Shaughnessey & Palmer, 1989; Russell, 2000; Warshaw, 1988). This is acquaintance rape (Black, 2011).^2^ Survivors are less likely to label their experience as rape when there is a lack of force employed by the attacker and a lack of resistance from the survivor (Wilson & Miller, 2016), both of which are more common in acquaintance rape. Because this type of attack does not match the stereotypical scenario, victims may rationalize their experience as not being rape (Kahn, Jackson, Kully, Badger, & Halvorsen, 2003; Littleton, Axsom, Breitkopf, & Berenson, 2006). Further misunderstandings may result from how consent is conceptualized and the conflation between consent (the only thing that defines rape) and wantedness (Peterson & Muehlenhard, 2007). Here we summarize scientific understanding of survivors’ and perpetrators’ conceptualization of consent and rape, linking this to sexual script theory. Subsequently, we focus on how people might interpret scenarios involving pleasure.

Legally, consent is usually defined as the person agrees to participate by choice, with the freedom and capacity to choose (Department of Justice, 1985; Home Office, 2003). This, therefore, means that consent can be given, but the act is rape if the consent was forced through threats, drug, or alcohol use (Kahn, Mathie, & Torgler, 1994; Koss et al., 1987; Shapiro & Schwarz, 1997; Testa & Dermen, 1999). Consent can be conceptualized as either present or absent (with sex in the latter case being rape). However, some researchers consider consent to be more of a scale (Panichas, 2001) and this may reflect how people view consent (Hills et al., 2020). Alternatively, typologies of consent suggest that it may reflect an internal state of willingness, explicit statements of willingness, and behaviors that may indicate willingness (Muehlenhard, Humphreys, Jozkowski, & Peterson, 2016).

In relationships, consent is typically provided through potentially ambiguous nonverbal behaviors (Beres, Herold, & Maitland, 2004) that are not always easy to read (Beres, 2007). Hickman and Muehlenhard (1999) categorize consent according to whether it is direct or indirect and whether it is verbal or nonverbal. There are many nonverbal cues to consent—for example, reciprocation in acts (and a lack of consent—for example pushing someone away) and these appear to be understood by many young adults (Beres, 2014). However, different behaviors communicate consent and these may not be universal. Indeed, participants describe their “need” for consent depends on a number of factors (Willis et al., 2019) including length of relationship (Humphreys, 2007), sexual experience of the individual (Humphreys, 2005), gender (Humphreys, 2007; Humphreys & Herold, 2007), type of sexual act (Hall, 1998), and timing within the sexual scenario (Beres, 2014). These outward behaviors indicative of consent may not always reflect the cognitive feelings behind them (Muehlenhard, 1996): In fact only a mild to moderate relationship between internal (feelings of consenting) and external expressions (actually showing that one consents) of consent has been demonstrated (Jozkowski, Peterson, Sanders, Dennis, & Reece, 2014). This can lead to problems with understanding if consent has been given (Hickman & Muehlenhard, 1999; Jozkowski & Peterson, 2013).

Consent is far more nuanced than can be briefly described in this short introduction. Firstly, consent could be considered a process. During a sexual event, people’s initial plan will change and develop (Beres, Senn, & McCaw, 2014). The activities that one consents to will also change during a sexual act, meaning that it is an ongoing process (Beres, 2014) subject to frequent changes. This means that there will be uncertainty and ambivalence around what activities one will consent to at the start of an encounter that can only be resolved through communication (Muehlenhard & Peterson, 2005).

To elucidate the problem with defining consent, Peterson and Muehlenhard (2007) described how there is a separation of “wantedness” and “consent” when considering the labeling of sexual encounters as rape. Wantedness reflects a desire to do something or believing that it is good (Peterson & Muehlenhard, 2007). Wantedness might influence consent, but it is distinct from it. For example, a person may want sex (because they are highly attracted to someone, for example), but not consent to it (because they are married, for example). Sexual acts can therefore fall into one of four categories: wanted and consensual; unwanted and consensual; wanted and non-consensual (rape); unwanted and non-consensual (rape).

Consenting to unwanted sex can occur for many reasons: to build intimacy, satisfy partners’ desires, flirtation, desiring pleasure, avoiding relationship tension, avoiding hurting partners’ feelings, maintaining a relationship, feeling obligated because of something the partner has done, or to control feelings associated with anxious attachment (Conroy, Krishnakumar, & Leone, 2015; Drouin & Tobin, 2014; Gilbert & Walker, 1999; Impett & Peplau, 2002; Koss et al., 1987; Muehlenhard & Cook, 1988; O’Sullivan & Allgeier, 1998; Shotland & Hunter, 1995; Sprecher, Hatfield, Cortese, Potapova, & Levitskaya, 1994; Tolman & Szalacha, 1999). Indeed, O’Sullivan and Allgeier (1998) found over a two-week period, 38% of participants in committed relationships consented to unwanted sex. People may also not consent to wanted sex, for reasons such as not having a condom and to wait until after marriage (Muehlenhard & Hollabaugh, 1988; Muehlenhard & Peterson, 2005; Muehlenhard & Rodgers, 1998). Peterson and Muehlenhard (2007) found that 18.9% of their participants who had been raped actually wanted the sexual act to some degree but did not want the consequences (for example, they were aroused due to flirting, but did not want to be raped). Real and mock jurors tend to ignore lack of consent if wantedness is present (McHugh, 1996), implying the presence of wantedness mediates judgments of rape. Peterson and Muehlenhard’s data imply that pleasure may be a separable construct from wanting.

In Peterson and Muehlenhard’s (2007) definition of wanting, they include a desire for something to happen and to believe that it is positive or pleasurable. These two aspects reflect distinct constructs. Physiological pleasure is typically defined as how enjoyable intercourse is in terms of sexual arousal (Basson, 2001) and is an autonomous mechanism that creates sexual arousal at a subcortical level (Levin & Van Berlo, 2004). While Tolman and Szalacha (1999) suggest feelings of pleasure are a reason for wanting sex, other researchers have indicated that pleasure is not the only reason for wanting sex (Cain et al., 2003). People often want and consent to sex not because it will bring pleasure, but because it may help build a relationship or they are trying to get pregnant, for example. Within relationships, by managing a partner’s needs, it is possible a person might find pleasure in sexual intercourse that was not consensual (Basson, 2001, 2005). Physiological pleasure is possible from unwanted and non-consensual sex as it is fundamentally a physiological process, despite subjective emotional states being described as “anxiety-provoking,” “feared” and “unpleasant” (Basson et al., 2003; Levin & Van Berlo, 2004; Suschinsky & Lalumière, 2011; Van Berlo & Ensink, 2000). Indeed, such scenarios might make the victim feel more guilt and blame (Lofgreen, 2014). Furthermore, pleasure affects how individuals label an experience as rape and how much psychological distress they feel afterward (Basson, 2005; Kilpatrick, Veronen, & Resick, 1982) and strongly predicts blame attribution and high judgments of rape (McCaul, Veltum, Boyechko, & Crawford, 1990).

In a recent study by Hills et al. (2020), a set of sexual scenarios between acquaintances were created that systematically manipulated consent, wantedness and pleasure. These were given to participants to rate how much they reflected the participants’ own definitions of rape (using a scalar judgment or a binary decision). Surprisingly, only 65% of non-consensual scenarios were rated as rape (with the context that 9.35% of consensual scenarios were considered rape). Indeed, consent, wantedness and pleasure all affected the ratings of rape—the absence of any feature led to higher judgments of rape (despite the only requirement for rape decisions being a lack of consent). Further, ratings of rape in non-consensual scenarios were moderated by wantedness and pleasure, whereby the lack of these features increased the chance that the scenarios would be rated as rape. Critically, there were few gender differences found in these data: males used pleasure to moderate non-consensual and unwanted scenarios, whereas women did not show this three-way interaction. While only consent matters to the legal definition of rape, Hills et al.’s participants conflated wantedness with consent and used subsequent pleasure to moderate their judgments.

The link between wanting, consent and pleasure relates to the heteronormal sexual script. Traditional heteronormal sexual scripts involve men initiating sex more than women and seeking it out (Tolman, Kim, Schooler, & Sorsoli, 2007). This is consistent with the masculine and hierarchical rape culture, particularly in fraternities, where women and sex are seen as goals (Jozkowski, Marcantonio, & Hunt, 2017). This means that women act as gatekeepers to sex (Cannon, Lauve-Moon, & Buttell, 2015). Men must actively seek out and obtain sex by asking for it (Kitzinger & Frith, 1999). However, verbal communication in sexual scripts is not typical. This creates a power imbalance in sexual scripts with men more dominating and active participants and women the passive recipients (Sanchez, Fetterolf, & Rudman, 2012). Indeed, women are not supposed to actively express their sexuality within this traditional heteronormal sexual script (Wiederman, 2005). Gendered sexual scripts can become internalized such that both men and women consider it the norm (Ward, 2003). Indeed, females who have internalized this sexual script have been found to be more likely to engage in unwanted sex (Bay-Cheng & Eliseo-Arras, 2008). These sources of evidence provide avenues for future intervention strategies.

While the data presented by Hills et al. (2020) are interesting, the quantitative results do not explain *why* participants used other features in rating sexual scenarios as rape and whether they used sexual scripts to interpret the scenarios. Indeed, all Hills et al. can provide is ratings that may or not link to actual behavior: qualitative responses are more likely to provide better links to attitudes and behaviors. Previous work indicates that consent and wantedness tend to be conflated (Peterson & Muehlenhard, 2007); however, the impact of pleasure is difficult to explain as the conflation between consent and pleasure has not been extensively researched.

The present study aims to investigate why university students rate sexual scenarios between acquaintances as rape or not. University students are at a high risk for experiencing sexual assault and acquaintance rape (Bachar & Koss, 2001; Daigle, Fisher, & Stewart, 2009) especially first-year undergraduate students (McCluskey-Fawcett, Berkley-Patton, Towns, & Prosser, 2001), with 62% of recent graduates reporting that they had experience sexual violence (Revolt Sexual Assault and The Student Room, 2018). Using the scenarios devised by Hills et al. (2020), we investigated participants’ subjective opinions of sexual scenarios. In these scenarios consent, wantedness and pleasure were varied systematically. Participants read these scenarios and provided their reasons as free-text responses for why they believed the scenario indicated rape or not.

We applied content analysis to these as an appropriate tool to analyze written communication (Bardin, 1977; Hsieh & Shannon, 2005). This involves exploring the data in order to find how many times particular categories are mentioned. The intention is to identify patterns in the circumstantial, objective and/or subjective aspects participants took into consideration when rating the situations as rape and/or distressingness. Specifically, this content analysis approach allows us to analyze the different variables involved with the use of frequencies. Utilizing exploratory content analysis (i.e., by not having pre-determined categories) also ensures the subjective richness of the data is not lost and might lead to the creation of new theories (Krippendorff, 2013).

Two versions of the study were run. The purpose of this was to include a replication of the findings to explore the consistency of the results. The second was to examine the impact of the perspective taken within the scenarios had on how participants interpreted the scenarios. In Study 1, participants freely read the scenarios and were asked to imagine themselves in the scenario. In Study 2, participants were either instructed to imagine themselves as the initiator of the sexual encounter or as the subject of the sexual encounter.^3^

## Study 1

### Method

#### Participants

An opportunity sample of 131 (94 female, age range 18 to 36 years, mean age 22 years) psychology undergraduates took part in this study in return for course credits. These were recruited from two universities in the UK, and 80% of them were studying in their first year. One hundred and fourteen self-reported they were White. Participants were recruited via an online advertisement that asked participants to take part in a study “exploring people’s attitudes to sexual scenarios” and informed that some of the scenarios would depict non-consensual sexual encounters and anyone with experience of rape was advised not to take part. The main reason why more women took part in this study is likely to reflect that the advert contained the taboo word “rape” and that psychology departments in the UK consist of 85% female students, and this is where our sample was recruited.

Table 1 highlights the characteristics of the participants and the universities from which they were recruited. The main differences between the universities is that Anglia Ruskin University (ARU) has a higher proportion of non-White and female students than Bournemouth University (BU) and is at an urban campus compared to a suburban campus, respectively. Testing at two universities allowed us to see if the findings replicated across institutions. All other characteristics are similar to other UK universities.

**Table 1:** Participant characteristics and comparison of the universities from which they came from

	Bournemouth University	Anglia Ruskin University	Bournemouth University (Study 2)
Participant *N*	95	39	155
*N* female	67	27	143
Mean age	21.6 years	25.1 years	20.21 years
Age range	18–26	19–36	18–34
*N* first-year undergraduates	90	18	139
*N* white	91	17	150
*N* single	14	26	
Campus type	Suburban campus	City campus	
University age	1992	2002	
University group		Million +	
Number of students	17,880	14,996	
Student demographic			
% Female	55%	62%	
% Under 21 years old	42%	35%	
% White	78%	61%	
% Disabled	14%	13%	

Data sourced from Bournemouth University’s Annual Report for Equality and Diversity and Anglia Ruskin University’s Campus Snapshot

#### Materials

Twenty-four vignettes developed by Hills et al. (2020) were used for this study: 12 were consensual and 12 were non-consensual: They are available at 10.1037/xap0000221. The scenarios were constructed based on the guidelines presented by Barter and Renold (1999). The scenarios were based on plausible scenarios developed from the “Reasons for Wanting Sex and Reasons for Not Wanting Sex Subscales” of Peterson and Muehlenhard’s (2007) Wanting Questionnaire. These vignettes have been extensively pretested to ensure that they adequately demonstrate the three variables of interest for this study (consent, wantedness, and pleasure): further they were matched for the clarity with which they displayed the key feature and clarity of writing to ensure they had internal validity. They were comparable in length and structure and content to those asked in a large scale YouGov survey (YouGov, 2018). When asked if they show consent or not, participants are able to determine this accurately (see Hills et al., 2020). Each is a short (two- to three-sentence) hypothetical sexual scenarios between acquaintances: None of them described any explicit imagery or words but rather focused on the build up to the sex (sex was not defined within the scenarios to allow participants to interpret this within their own script). Sex was mentioned insofar as the characters had sex without graphic detail. They were written in the second person and gender neutral. They were written in such a way that they allowed for sufficient individual interpretation but were easy to understand, using everyday simplistic language. These vignettes allow for an understanding of core elements within decisions of what constitutes rape. Further, it allows us to directly explore the sexual script that our participants might have. For example, a consensual, unwanted, not pleasurable scenario would be:

You feel uncomfortable about your body; therefore, you don’t want to engage in sex. However, when your partner suggests to have sex, you agree. Nevertheless, the experience is not enjoyable to you.

Because there were many disparate reasons for not wanting sex (such as not having a condom or because the person in married), these scenarios were varied in terms of content. Further, these vignettes capture the variability in how consent can be coerced and forced. We were careful to ensure that there was no phrase that stated consent was given during the non-consensual scenarios. The words “agree” and “consent” were clear in consensual scenarios and were never present in the non-consensual ones. While this means they were not completely systematic, it means they better reflect sexual scenarios between acquaintances. Further, for the content analysis, this variability is largely ignored as we attempt to extract underlying themes that crossover the variability: We explore the general themes that lead people to decide if a sexual scenario is rape or not. While there is not a one-to-one relationship between vignette responding and actual behavior, well-constructed vignettes do give an indication of how participants understand situations (Eifler, 2007).

#### Procedure

The study was granted full ethical approval by the Research Ethics Panels at both universities. The study was run online in order for the participants to feel comfortable giving open responses: Their anonymity was ensured. After providing consent, participants read through the first vignette at their own pace. They were asked to “imagine you experience the described situations.” This allowed participants to apply their own gendered sexual script to the vignette. Participants wrote free-text comments regarding the situation explaining why they believed the situation displayed rape or not. We used the term rape as we were specifically interested in participants understanding of the legal term “rape” rather than the more general “sexual assault.” This was chosen as we did not anticipate our participants understanding that in UK law only men can rape (indeed, evidence suggests few UK citizens are aware of this fact, YouGov, 2018). The participant completed each vignette in turn and were not permitted to go back to a previous vignette to change their answers. The order of the vignettes was randomized across participants. Participants completed the survey at their own pace meaning that participants could leave the browser open and return to the task after leaving it. We believe 10% of participants did this as they took over 2 h to complete the vignettes. Excluding those participants, on average, participants took 29 min to complete the study. The amount of text for the last eight vignettes was 85% of the first eight vignettes indicating that there was only minimal fatigue in the study. Further, because the vignettes were presented in a random order, this variation was split across all conditions equally. Once participants had completed all 24 vignettes, they were thanked and debriefed.

#### Data Analysis

Participants’ free-text comments (as to the reasons for why they thought the scenarios were or were not rape) were analyzed using content analysis. This common technique was used to reduce large quantities of data into a few meaningful categories. We followed the procedures of Elo and Kyngäs (2008). The unit of analysis was phrases (as opposed to single words, full sentences, or themes) as this maintains the context of the response and prevented fragmentation. The data were open coded (categorized) before being recoded into higher-order groups. This reduces the number of individual categories into more manageable groups. The groups were then named and described. This was done by the second and third authors under the supervision of the first author.

Triangulation occurred with this process being repeated blind by a research assistant. The research assistant did not have access to any information that the second and third authors produced. The theme table created was consistent with that constructed by the second and third authors (with subtly different names for the groups). The first author checked that these were consistent and developed a code book detailing the open codes and their links into higher-order groups.

Once the data were coded into discrete categories, frequencies were devised. This was done for each condition independently and is shown in Table 2. Throughout this analysis, we explored whether there were any differences across institutions or across participant gender. Any differences are highlighted here.

**Table 2:** Summary content analysis (full analysis presented in supplementary table S1) highlighting the category, a description and how it is used across conditions, and the number of mentions in the participants’ responses in their reasons for appraising situations as rape or not (% male responding with this)

Category	Description	Mentions							
		CWP	CWnP	CnWP	CnWnP	nCWP	nCWnP	nCnWP	nCnWnP
Consent	Participants frequently used consent to appraise the situations. This was sometimes qualified by the presence of pressure or force. There was clearly some difference in whether participants felt scenarios displayed consent indicating some misunderstanding of consent	193 (30%)	174 (35%)	180 (34%)	180 (29%)	137 (24%)	179 (45%)	118 (41%)	125 (35%)
Wanting	While a key variable, participants did not use the category wantedness when appraising these scenarios and quite often conflated wantedness with consent	7 (5%)	11 (0%)	21 (40%)	7 (83%)	19 (20%)	12 (50%)	14 (50%)	20 (33%)
Pleasure	Pleasure was used to appraise situations as rape, especially when the pleasure was inconsistent with consent. Some participants did highlight that pleasure is irrelevant when judging rape	34 (35%)	20 (40%)	17 (36%)	23 (16%)	60 (19%)	62 (21%)	58 (52%)	43 (30%)
Both	With the more consensual and wanted scenarios, participants used the word “both” to describe the interaction. This did not happen for non-consensual ones	249 (26%)	69 (22%)	18 (18%)	59 (19%)	10 (30%)	12 (67%)	9 (22%)	3 (0%)
They	Participants used more distancing phrasing in the less pleasant scenarios	5 (100%)	66 (31%)	91 (31%)	60 (32%)	45 (26%)	56 (35%)	47 (47%)	66 (33%)
Force	Force, pressure, coercion was used by participants to appraise the situations. This created much variability in how it was used with some participants indicating pressure is acceptable (especially in relationships), whereas others indicated that it was never acceptable	0	22 (33%)	47 (38%)	16 (17%)	16 (41%)	37 (38%)	120 (34%)	53 (47%)
Communication	Participants did consider the nature of communication between partners affected whether situations were considered rape. Some participants indicated that partners need to say “stop”; otherwise, it is not rape	1 (0%)	42 (14%)	15 (0%)	22 (31%)	1 (0%)	18 (44%)	6 (83%)	6 (0%)
Consequences	The stress caused by the situation was used by a few participants to appraise the situation as rape	7 (0%)	6 (0%)	9 (0%)	9 (0%)	9 (0%)	5 (50%)	5 (0%)	3 (0%)

These mentions include participants stating that the presence or absence of the category mattered and whether the category was relevant to their decision

Condition labels are *C* consent, *W* wanted, *P* pleasurable (with a lowercase *n* indicating it wasn’t included in the vignette)

## Results and Discussion

The content analysis indicates that consent was used by our participants to judge whether a scenario described rape more frequently than any other category. Consent (or lack of) was mentioned more frequently in the consensual scenarios (727 mentions) than the non-consensual scenarios (559 mentions). Participants (16%) used the phrase “giving in” to highlight that as soon as consent is provided (55 mentions); even if “dubious” (8% of participants), it is still consent. The notion of how consent was reached did not matter to all participants but did matter to some. This is highlighted by such quotes as “consent is still given” (BU, M, 21). In 8% of cases, participants also indicated that without clear communication of a lack of consent, then consent was provided and these situations are not rape highlighting a misunderstanding of rape. Interestingly, this opinion was equally made by both females and males.

Specifically, consent was qualified by the presence of pleasure by 9% of participants. When pleasurable, many participants said that they lowered their ratings of rape even if the scenario did not describe a consensual scenario. Some (4% of participants) suggested that the enjoyment of the situation will reduce distress and mean the situation is not rape. Indeed, some (2% of participants) considered that subsequent pleasure meant that retrospective consent could be given or assumed. 6% of the participants indicated that non-consensual (but wanted and pleasurable) situations were rape with “dubious consent:” They acknowledged that the subsequent pleasure actually made them feel better after the sex and made them less inclined to think it was rape:

How you would feel would be greatly effected by whether you did actually regret it later. But the person should still have respected your wishes not to have sex in the first place. (ARU, M, 36)

Participants (1%) did indicate that non-pleasurable situations should stop (otherwise, they might be considered rape), but only if the partner was aware. This relates to the category of communication. A total of 27% of participants indicated that unless the partner said “stop” it was not rape. Other participants (9%) indicated that the active partner ought to be aware of their partner’s feelings, but that they may not be “mind-readers” (BU, F, 19). Some suggested scenarios without wantedness or consent were negative and involved an uncaring partner who should have more awareness of their partner’s feelings.

It can only be classed as rape if you tell your partner to stop and they don’t if they cannot tell that you are not enjoying the situation how are they to know unless you say something. (ARU, F, 19)

Pressure, force, and coercion were mentioned by 39% of participants in their appraisals of these situations. This category produced highly variable responses and interpretations. Several participants (3%) indicated that “Sometimes a little push is needed to convince someone” (BU, F, 20). Indeed, another participant indicated that while physical pressure would lead the situation to be considered rape, “constantly suggesting it,… then definitely not rape” (ARU, M, 25). Several participants (5%) also did not consider non-consensual situations as rape because they often occurred within relationships, with one participant indicating that because consent had been given before, it can be assumed again. This is a typical rape myth. As a male from BU (20) put it, “This is fairly common in relationships, from both parties.” In these situations, many participants confused wantedness with consent.

For some, the level of coercion required to define a situation as rape was violence or verbal abuse. There were some participants who strongly held on to the point that consent is all that matters:

But if “you give into it” means you agree and don’t tell them to stop, then it is not rape. (BU, F, 19)

Some of our responses (6%) were also indicative of victim blaming. One participant indicated:

You need confidence to say no- and also be with someone who respects you enough to accept your answer when you say no. (ARU, F, 28)

This statement puts blame onto the victim somewhat, for not being with the right person and for needing to have the confidence to say “no.” This notion was repeated by 7% of participants indicating they might feel guilty if they had initially led their partner on and then changed their mind. Several participants reported that such situations were not rape because of the victim’s behavior.

What matters is how you acted/what you said just before sex was initiated. If you refused then this is rape. (ARU, M, 25)

The language used across the types of vignettes also differed suggesting much more agency in the most pleasant scenarios than in those that were less pleasant. This may indicate an aspect of psychological distancing from the participants—they may be attempting to make the negative scenarios appear to be happening to other people, whereas the positive scenarios happen to themselves. It was easier for participants to imagine themselves in the positive scenarios rather than the negative ones. Our final two themes highlight this. In the consensual, wanted and pleasurable scenarios, participants used the term “both” much more (249 mentions, referring to both parties in the sexual act) than in any other scenario combined (180 mentions).

We both agreed to have sex. (BU, F, 20)

Indeed, it seemed like there was a trend for the use of the term “both” to decrease the more the scenario became unpleasant. The phrasing used indicated that there was something mutual about the situation, whereas, when the situation was less pleasant, the phrasing did not implicate mutual participation. The converse was true with the use of the active voice: participants used the third person (“they”) much less in the consensual, wanted, and pleasurable (5 mentions) scenarios than the other scenarios (45 to 91 mentions).

They didn’t want to have sex and were uncomfortable. (BU, F, 18)

The use of the word “they” rather than “we” highlights that participant was writing about someone else or an event happening to somebody else. We interpret this as a way of distancing themselves from the imagined scenario.

Throughout this analysis, we have incorporated comments from both females and males. While males and females will interpret the scenarios differently, the scenarios did allow for participants to imagine themselves in the situations. Participants would use their gendered lens to read these scenarios. As one female from BU put it:

struggling to answer these questions from a girls perspective, but would say that its hard to have sex with a male if they didnt want to, there shouldn’t be pressure though. (BU, F, 20)

This comment contrasted to comments indicating how several of the scenarios are “normal” in relationships (7% of participants said this).

Because they are in a relationship and the partner agreed eventually. (BU, F, 22)

Additionally, by not making the detail of the sexual activities explicit (whether vaginal, anal, or oral), it did allow both female and male participants to imagine themselves in the scenarios. Indeed, there were few thematic differences across gender in this study similar to Hickman and Muehlenhard’s (1999) findings. There were no language differences across participants in how often females and males used the first person and active language. This indicates that females and males did read these scenarios similarly. Further, Hills et al. (2020) did not indicate gender differences in the way the scenarios had been rated.

The main gender differences that we observed were that no male participant considered the emotional consequences (such as how it would make the subject of the scenario feel) during their reading of the scenario. This is consistent with participants reading the scenarios through their gendered lens: Males are much less likely to report being sexually assaulted and raped than females. This means that they are less likely to need to consider the emotional, physical, and psychological consequences of sexual assault and rape. Our data reflect this aspect of sexual assault. Our second gender difference is consistent with the previous observation. Our male participants made fewer comments than our female participants. Overall, male participants mentioned things less frequently than female participants, suggesting that they think less about sexual assault.

Finally, it was worth noting that there were no discernible differences in themes across the institutions. We were able to draw highly similar quotes from both female and male participants for every theme. Indeed, the results seem to replicate across these institutions indicating that rape culture (Herman, 1988) is unlikely to be significantly different across these institutions.

## Study 2

Study 1 indicated that students drew on consent, wantedness, and pleasure when constructing their appraisal of sexual scenarios as to whether they represent rape. One weakness of Study 1 was that participants were instructed to “imagine you experience the described situations.” Given that two people were always described in the scenarios—the initiator and the subject—participants could have imagined themselves in either position. We might expect that our female participants imagining themselves in the subject position more than the initiator position because of gender roles and sexual socialization (Abrams, Viki, Masser, & Bohner, 2003; Buddie & Miller, 2001; Jozkowksi & Peterson, 2013). However, because the scenarios were written in a gender-neutral manner, all participants could have imagined themselves in either position.

Taking the perspective of the subject (victim) can result in rape minimization (David & Schneider, 2005) or increased empathy for other victims of rape (Weir & Wrightsman, 1990), encourage supportive behavior (Anastasio & Costa, 2004; Baker, 2015) and enhance altruistic behavior (Davis, 1996). Conversely, asking the participants to imagine themselves as the initiator, they might reappraise the sex as less unpleasant. We can base this supposition on Beres’ (2010) findings that when participants are asked to complete a narrative in which their partner initially refuses sex but they then accept it—scenarios of playfulness, convincing and coercion were far more commonly described than rape. As such, we re-ran the study in which participants were asked to take on either the perspective of the subject or initiator.

## Method

The methods were identical to Experiment 1 except the instructions (to imagine themselves as the initiator or the subject) and the phrasing within the scenarios. Scenarios were revised for those given the initiator’s perspective (see Hills et al., 2020). These changes were minimal and simply adjusted who was active in the scenario. For example:

You/Your partner feel[s] highly aroused about a possibility of having sex. However, when your partner/you tries to initiate having sex you/your partner reject because there are no condoms. Your partner/you pin[s] you/your partner down and forces you/your partner to have sex regardless. You/Your partner find your partner’s/your behavior appealing and attractive in the situation and enjoy the sex.

Participants were a different set of 155 (143 female, age range 18-57 years, mean age = 20 years) students recruited from Bournemouth University who had not taken part in Study 1. Participants were randomly allocated to read the vignettes from the subject’s or the initiator’s perspective. Because there were so few males in Study 2, we were unable to analyze the results by gender. Nevertheless, the results from Study 1 seem to suggest very specific gender differences in the resultant themes. The analysis was conducted by the second author initially and verified by the first author.

## Results and Discussion

The results of Study 2 were highly consistent with Study 1. Consent was mentioned more than any other theme. However, there was significant disagreement between participants regarding whether the scenario showed consent or not. Several participants inferred consent since the participants wanted or engaged in sex. A total of 26% of participants viewed “giving in” as being consent, whereas 13% indicated this was “coerced” consent, so not true consent (Table 3).

**Table 3:** Summary content analysis (full analysis presented in supplementary table S2) for Experiment 2 highlighting the category, a description and how it is used across conditions, and the number of mentions in the participants responses in their reasons for appraising situations as rape or not

Category	Description	Mentions							
		CWP	CWnP	CnWP	CnWnP	nCWP	nCWnP	nCnWP	nCnWnP
Consent	Many participants used consent as a key guiding factor when appraising the scenarios. There was some disagreement about whether scenarios represented consent or not, and several participants indicated a change in consent during the scenarios and that giving in may mean consent was offered	136	92	90	90	74	93	50	58
		134	97	112	111	73	105	74	86
Wanting	Wanting was identified as a reason to appraise the situations as rape or not. Wanting was often conflated with consent and used synonymously	2	2	26	18	15	12	9	7
		12	12	33	35	20	26	15	35
Pleasure	Pleasure was used by many participants to appraise the situations. When the situation was pleasurable, it often mitigated the lack of consent for some participants. Some participants, however, did feel that pleasure was irrelevant	26	16	8	17	28	13	19	13
		42	19	31	28	30	36	48	32
Both	For the more consensual and wanted scenarios, participants made reference to “both” partners. This was not the case in the non-consensual scenarios, indicating an awareness that the scenarios were not for both partners	97	46	12	3	7	1	0	0
		123	40	16	6	5	0	1	0
They	Psychological distancing was more present in the initiator conditions than the subject conditions, as indicated by the use of the third person tense	1	24	26	25	25	27	19	21
		3	37	90	91	47	69	60	91
Force	While there was disagreement about whether force or pressure led to rape and where the line was drawn between flirting and coercion, force was a contributing factor to the appraisals	4	5	26	16	62	36	46	42
		1	10	22	29	70	39	76	93
Communication	Many participants indicated that participants need to communicate their displeasure or lack of consent more frequently to ensure the situation is not rape	0	50	5	23	3	3	0	0
		0	34	15	19	2	5	1	1
Consequences	A few participants considered the potential distress in their judgments of rape	2	10	9	9	7	13	3	6
		3	7	7	4	3	3	6	5

These mentions include participants stating that the presence or absence of the category mattered and whether the category was relevant to their decision

Top value in each cell represents the subject position and the bottom value represents the initiator position

Condition labels are *C* consent, *W* wanted, *P* pleasurable (with a lowercase *n* indicating it wasn’t included in the vignette)

Whether participants felt a lack of consent led to judging a scenario as rape did depend on whether there was resulting perceived pleasure from the scenario: in 31% of non-consensual scenarios, pleasure was described as a justification. A few participants (3%) suggested that the enjoyment afterward implied that consent was retrospectively given. One participant indicated that it was “lucky” the partner enjoyed the sex:

It’s a bit stressful because the partner could have gone either way once being persuaded, but luckily they enjoyed it, so even though it was slightly forced by one partner, they were only initially bored and uninterested rather than not wanting intercourse. (BU, F, 20)

This implies they felt the enjoyment counteracted the lack of consent: without pleasure it would be considered rape. Nevertheless, the finding that pleasure played a big role in participants’ appraisals of these sexual scenarios is novel and important.

Wanting was often conflated with consent by our participants (22%). This indicates that there is a lack of understanding that people can want sex and not consent to it. Indeed, it seems that 4% of our participants could not “understand the context” of the scenario, whereas 7% indicated it was “normal in relationships.” This highlights unique and highly variable experiences of sex within relationships. Some people were more aware of consenting to unwanted sex than others.

We also found that a significant portion of our participants appeared to blame the subject for “bad sex” (BU, M, 20), defined as unwanted or not pleasurable sex, or non-consensual sex. 16% of participants indicated that the partner should refuse or communicate their displeasure and/or their lack of consent:

Consent wasn’t given although there is nothing to say you tried to stop the intercourse. (BU, F, 19)

Indeed, one participant highlighted that the partner should continually refuse otherwise the scenario was not rape.

They refused but then gave in and do not continue to refuse, so it’s not really rape. (BU, F, 20)

This indicates that there was a significant portion of our participants that were engaging in victim blaming behaviors and demonstrating a lack of understanding of some of the potential consequences of individual traits (e.g., a lack of assertiveness) or emotions (e.g., trust, fear).

Finally, we observed that there was a great deal of psychological distancing in the language used by 18% of participants. This is despite the instructions to attempt to get into the mindset of the subject or the initiator in the scenarios. Participants, especially in the initiator condition (489 versus 168 mentions in the subject condition) and in the non-consensual scenarios, used the inactive third person tense (“they”) to describe the situation. This was interpreted as a way for them to distance themselves from the scenario and to give more responsibility to the subject of the scenario than themselves as the initiator. This occurred even though the instruction was to imagine themselves in the situation. This further highlights evidence of participants blaming the victim, especially if they engage in non-ideal behaviors. This was the only discernible difference in themes resulting from participants taking the subject or initiator position.

An additional noteworthy comment was made by a participant who indicated that:

It is definitely rape because I did not consent to have sex, but I find this less rapey than if it was forced upon me by a stranger. (BU, F, 21)

This comment is consistent with previous work indicating that people consider acquaintance rape as less like rape and less distressing than stranger rape (McGregor, Wiebe, Marion, & Livingstone, 2000), though this does not match the experience of those who have experienced acquaintance rape (DiVasto, 1985; Finn, 1995; Koss & Burkhart, 1989; Koss, Dinero, Seibel, & Cox, 1988; Yeater & O’Donohue, 1999).

The results replicate and extend those of Study 1. Consent, wantedness, and pleasure affect how a sexual scenario is appraised. Our data support McCaul et al.’s (1990) assumption that the introduction of pleasure in a scenario that legally defines rape predicts victim blaming and consequent low labeling of the scenario as rape.

Our analysis revealed participants repeatedly referenced that aggressive rape scenarios were more deserving of higher rape scores, consistent with rape myth research regarding what constitutes a “real rape” (Bohner, Eyssel, Pina, Siebler, & Viki, 2009; Hockett et al., 2015). Interestingly, 9% of participants integrated aggressive terminology into the vignettes, potentially molding the situation into their accepted belief of what constitutes rape. Research suggests people “fill in the gap” in rape cases to justify their answer to fit internalized rape stereotypes (Krahé, Temkin, Bieneck, & Berger, 2008).

## General Discussion

In both our studies, we found that consent, wantedness and pleasure all affected how participants appraised rape in sexual situations despite consent being the only thing that is required for a sexual scenario not to be rape. Lack of consent was the biggest factor in determining whether a situation was perceived as rape with many participants (19%) stating in free-text responses consent was the only thing that mattered. However, wantedness and pleasure also influenced whether participants considered the situation as rape, despite neither being part of the definition of rape. This has important implications for the development of education packages and support services: If a survivor of rape is thought to want sex and get pleasure from it, our data indicate that this will less likely be perceived as rape, despite clearly being such and this may lead to negative unwanted circumstances. It has been found that jurors ignore lack of consent if wantedness is present (McHugh, 1996). This is further evidence that suggests it is vital to understand laypersons’ perceptions of sexual assault because they are the ones present when it happens, contribute to victimization of survivors of sexual assault, responses to victims and jury decision making (Angelone, Mitchell, & Smith, 2014).

The use of additional information in judging whether scenarios represent rape and some of the comments made indicate rape myths and victim blaming were present in our student sample (e.g., Aosved & Long, 2006; Bohner et al., 2009; Burt, 1980; O’Donohue, Yeater, & Fanetti, 2003): In particular, the myth that forced sex is justified if the victim appears to want sex prior to refusing (Payne, Lonsway, & Fitzgerald, 1999). This finding fits with earlier reports that sexual assault is more likely if the perpetrator feels that the victim wants sex and is leading the perpetrator on (Abbey, McAuslan, Zawacki, Clinton, & Buck, 2001; Malamuth & Brown, 1994; Muehlenhard & Linton, 1987). The former is indicative of abuse during relationships: Most sexual coercion occurs within relationships or by acquaintances (Baum & Klaus, 2005; Koss et al., 1988; Parrot & Bechhofer, 1991; Tjaden & Thoennes, 2006). One of the most alarming findings from our study is that many of our participants thought that “giving in” was always akin to consenting.^4^ This is evidence for rape myth acceptance in some of our participants. This work implies that recent campaigns and sex education to raise awareness of rape and rape myths have not reached our participants. Clearly more work is required to educate the public regarding rape and sexual coercion and the importance of wanting being separate from consenting.

Our free-text responses indicate that many scenarios led to ambiguity with some people interpreting consent from verbal means and others interpreting consent as “giving in.” Giving in may well reflect the heteronormal sexual script in which women are considered to be the gatekeepers for sex (Crawford & Popp, 2003). This means that women are supposed to act chaste and not overtly show their sexuality (Interligi & McHugh, 2018) that in turn means they have to be chased, encouraged and potentially coerced (Littleton & Axsom, 2003). Similarly, in the masculine culture, when men press women’s boundaries in order to have sex and succeed, this means that they “score” (Marks & Wosick, 2017). In our study, men and women used the phase “giving in” a roughly equivalent amount of time (11% of men and 9% of women used this phrase), indicating the heteronormal sexual script is held by both females and males. This finding suggests that, within relationships, open discussions about what consent is and how to give it may lead to healthier and less distressing interactions. Such a finding has important implications for relationship counseling. Both partners need to want sex and give consent to maintain a healthy relationship, assuming other power dynamics are controlled.

The results here indicate our participants (university students) show a fairly tolerant attitude toward acquaintance rape and that rape scripts are common in student populations (Chng & Burke, 1999; MacNeela, Conway, Kavanagh, Kennedy, & McCaffrey, 2014). Student populations typically have different acceptance of social norms and risky behavior thus are more accepting of rape myth supporting attitudes (Aronowitz, Lambert, & Davidoff, 2012). An alternative interpretation is that our participants might be using defense mechanisms to appraise the situations as not rape. This is an important point regarding how survivors of these and similar (non-consensual) situations should be treated. While it may be a defense mechanism not to label such situations as rape (Breh & Seidler, 2007), these data clearly indicate that people still consider them distressing. Therefore, it might be more appropriate to label these situations as rape in order to begin to deal with the situation emotionally (Botta & Pingree, 1997; Kahn et al., 2003). Indeed, in most counseling settings, dealing with trauma (emotional or physical) requires acceptance of it (Gray, Koopman, & Hunt, 1991; Kübler-Ross, Wessler, & Avioli, 1972; Maciejewski, Zhang, Block, & Prigerson, 2007; Prigerson & Maciejewski, 2008). Saying this, there are clear narratives presented by survivors of such situations who do not want to label their experience as rape (Peterson & Muehlenhard, 2007).

Taken together, these results indicate participants do not simply use consent to appraise whether a depicted sexual scenario between partners is rape. Many participants use as much information as they are provided to judge whether a sexual scenario represents rape or not. This work is important in developing educational packages for the public and jurors. A lack of acknowledgement of the occurrence, severity and existence of acquaintance rape by jurors and judges (Gamble, 2014; Wilson & Leith, 2001) may be partially responsible for the low conviction rate (approximately 6% of reported rapes result in a conviction; Rape Crisis England & Wales, 2017; Temkin & Krahé, 2008). It is therefore important to understand how people (who could be jurors) appraise sexual scenarios that occur between acquaintances (Angelone, Mitchell, & Grossi, 2015). Our findings have important implications for the criminal justice system. If potential jurors are under the impression situations that are originally wanted or have pleasure are not rape (even though consent is not present), then potential rape perpetrators may not be convicted. Further, jurors will use all the surrounding context in judging whether a scenario is rape or not, when the only question is whether the perpetrator reasonably believed that consent was given. It is therefore imperative to educate jurors and criminal justice practitioners regarding these findings. In addition, it is of note that even if consent is dubious as the result of “giving in,” many participants considered it as consent because of an “uncaring partner” or a lack of “explicitly saying no.” This highlights a need to improve education regarding consent given to university students.

Our results provide further evidence that Beres’ (2014) assertion that sexual violence prevention needs to be centered around a message of “get consent” rather than the more traditional “no means no.” The benefit of this is that it moves the responsibility from the person who was raped to the person who raped (Pineau, 1989). Our results highlight the fact that this message has not been fully received since a significant number of the participants thought a repeated obvious statement of refusal was needed to prevent rape rather than the absence of consent.

### Limitations

As with all studies, the present work here has several limitations. Firstly, the changing social context around rape culture (associated with the heteronormal rape scripts held by university students) might make these results temporally and locality specific. The #metoo movement, for example, rapidly grew from the Tweet by Alyssa Milano in October 2017, leading to half a million responses in 24 h to making international mainstream news in a matter of weeks. The awareness of the issue of sexual violence was raised very quickly: Reports to the police increased by 26% in the U.S. (Seales, 2018). However, there is no evidence that the #metoo movement has led to significant changes in rape culture, suggesting a limitation of these kinds of studies in terms of generalizability.

By far the most significant limitation of the present study is the use of vignettes. While vignettes are a common method of research in qualitative research (Hughes & Huby, 2012), there is the potential that they do not link well to actual behavior (Carlson, 1996). This is especially the case when participants are asked to adopt the perspective of the characters in the scenarios and the scenarios do not match their typical behavior (Bettor, Hendrick, & Hendrick, 1995) such as would be the case in our initiator scenarios. Nevertheless, the benefits of vignette studies in obtaining information about sensitive topics (Finch, 1987) outweighs these negatives.

Our work was conducted at two universities in the UK: The results were highly similar across these institutions potentially because the cultures are not vastly different across the universities. Potentially, the increased diversity and larger proportion of women at Anglia Ruskin University could have led to different results to those obtained from the more homogenous population at Bournemouth University (given that egalitarian cultures have less of a rape culture than non-egalitarian ones, Barnett, Sligar, & Wang, 2018; Williams, Sawyer, & Wahlstrom, 2012). Since this was not observed and future research might explore more diverse and different university cultures. Indeed, future research should examine these questions in more ethnically and sexuality diverse samples. Our research suffers from a similar issue to many research papers do: The sample tested was predominantly heterosexual White middle-class people. There is no reason to believe that the attitudes expressed by our participants would replicate into non-White and non-heterosexual samples.

While this work was conducted solely on university students, as these are at a high risk for experiencing sexual violence (Cantor et al., 2015), we are aware of how this work might be applicable more broadly. Throughout this discussion, we have implied other contexts where these findings might be applicable. Indeed, we have no reason to suspect that the findings here would be different in a non-student sample, suggesting the work has implications for the criminal justice system. Nevertheless, future work would need to directly explore these contexts to ensure generalizability.

Finally, in this study we used the term “rape” in our attempt to obtain data. This was chosen because we have a larger aim to educate the community. Further, we did not feel participants would understand the difference between rape and sexual assault because of a lack of education regarding these terms in the UK (YouGov, 2018). Nevertheless, this could be explored in future.

### Conclusion

These results, in concurrence with other studies, have many implications for the training of jurors in rape cases and in extending educational programmes for rape and sexual assault prevention. A primary outcome of the present findings is that there is a distinct lack of understanding of what rape is and what factors contribute to people’s interpretations of whether a scenario is rape or not. Perceptions and attitudes were highly variable. This, on its own, is a sufficient basis for education programmes. One theme that both females and males raised was the need for communication. While this theme included victim blaming ideas, it also included the idea that it is important to communicate in relationships. This offers a future aim of work in this field, to encourage more open discussions of consent in order that there could be more peer disclosures. This could challenge the traditional sexual scripts that seem to exist. There is evidence that the average juror tends to hold a heteronormal sexual script (Stuart, McKimmie, & Masser, 2019) that can lead to a bias in the way they interpret court proceedings (Rerick, Livingston, & Davis, 2019). One of our participants put it wisely: “Arousal does not mean consent” (BU, F, 21). Yet, many jurors do tend to believe the presence of pleasure meant it was not rape: i.e., the “she liked it” effect (Booth, Willmott, & Boduszek, 2017; McGregor, 2005). Jurors need to separate wantedness and pleasure from consent and ensure that all sex without consent are treated as rape and that unwanted but consensual sex is as damaging psychologically as non-consensual sex. Alternatively, the instructions given to jurors might need to be altered to ensure that their discussions are less affected by their own biases and rape myth endorsements. For example, in the UK, the Bench Statements (Maddison, Ormerod, Tonkin, & Wait, 2018) include judge’s direction to be made in rape cases aimed at dispelling certain rape myths. The present work could, therefore, be utilized in updating or improving juror instructions (in the U.S.) and judge’s directions (UK), given that their use, understanding and effectiveness remain a problem (Bain, 2018).

## Electronic supplementary material

Below is the link to the electronic supplementary material.

Supplementary material 1 (DOCX 50 kb)

## Footnotes

1 While there are differences in the definition of rape across jurisdictions, typically it is defined as intentional penetration of the vagina, anus, or mouth of another person without their consent (Department of Justice, 2012; Home Office, 2003). Due to the similarities in definitions across jurisdictions, we have used evidence from several regions that have similar definitions of rape and consent. Further, the issue of sexual assault in university campuses is a worldwide problem with many similarities across jurisdictions in terms of research findings. Where there are culturally specific findings or effects, we have made these clear.

2 Though some law enforcement agencies define acquaintances differently: the UK police define acquaintance rape when the offender and victim had interacted over more than 1 day.

3 The term subject and initiator were chosen rather than the typical objects (or survivors) and perpetrators as is typical in the literature because, in half of the scenarios, consensual sex (therefore no rape) was depicted. The term “perpetrators” has the connotations of a criminal or negative act, and we felt this should be avoided when describing consensual wanted sex.

4 Of course, in healthy relationships, it is conceivable that gentle persuasion is part of a sexual script and is actually wanted. However, the evidence on token resistance suggests (Sprecher et al., 1994) it is not as commonplace as our participants reports of “giving in” being akin to consent.
