# Supplementary material for: Understanding How University Students Use Perceptions of Consent, Wantedness, and Pleasure in Labeling Rape
Source: Arch Sex Behav. 2020 Jul 8;50(1):247–62. doi: 10.1007/s10508-020-01772-1 (PMC7878243; doi:10.1007/s10508-020-01772-1)
Supplement: Supplementary file 1 — Supplementary material 1 (DOCX 50 kb) [file 10508_2020_1772_MOESM1_ESM.docx]

Table S1.

*Content Analysis tables for Study 1. Tables include content categories, the number of times the category was mentioned as being used to make judgements about rape, the number of times the lack of the category was used to make judgements about rape (% of male responses in this category), an explanation of how the category was used, and example quotes (with University affiliation - ARU=Anglia Ruskin University, BU=Bournemouth University – and participant gender – F=female, M=male).*

| ***Consent – Wanted – Pleasurable*** | | | | |
| --- | --- | --- | --- | --- |
| ***Category*** | ***Positive Mentions*** | ***Negative Mentions*** | ***Description*** | ***Example of Transcripts*** |
| Consent | 193 (30%) | 0 | Consent was mentioned by 61% participants | “Both in agreement” (BU, F)  “You both consent, definitely not rape” (ARU, M. |
| Wanting | 7 (5%) | 0 | 8% of participants mentioned that the scenarios were also wanted and consensual | “you both wanted it” (BU, F)  “I want to have the sex experience, and I agreed to have sexual intercourse, and I enjoyed it” (ARU, M) |
| Pleasure | 34 (35%) | 0 | 24% of participants highlighted that these scenarios were pleasurable, good, normal, or ideal. | “Sounds like consensual good sex to me. You want to do it and you both agree to it and enjoy it. and no indication that it was bad in any way.” (BU, F)  “enjoyment and agree” (BU, M) |
| Both | 249 (26%) | N/A | For these scenarios, 68% of participants highlighted that "both" partners were involved in the vignette. | “because you both have agreed” (BU, F)  “Both parties gave full consent. Definitely not rape.” (ARU, M) |
| They | 5 (100%) | N/A | Typically, participants did not refer to "them" or "their" consideration here. The language was much more active. |  |
| Force | 0 | 0 |  |  |
| Need for communication | 0 | 1 (0%) |  |  |
| Consequences | 7 (0%) | 0 |  | “Normal sexual encounter, I see nothing stressful about it and it's not rape.” (ARU, F) |

| ***Consent – Wanted – Not Pleasurable*** | | | | |
| --- | --- | --- | --- | --- |
| ***Category*** | ***Positive Mentions*** | ***Negative Mentions*** | ***Description*** | ***Example of Transcripts*** |
| Consent | 174 (35%) | 0 | Consent was mentioned by 61% of participants | “both consent” (BU, F)  “mutual agreement makes it not rape” (ARU, M) |
| Wanting | 5 (0%) | 6 (0%) | Participants (5%) highlighted that in these sexual scenarios, the sex was wanted. However, 5% of participants highlighted that the experience was not wanted. | “Engaging in sex is not rape, as you want to do it and give consent.” (ARU, F) |
| Pleasure | 0 | 20 (40%) | Participants (10%) highlighted that the not pleasurable nature of the sexual encounter affected their judgement. However, 8% of descriptions indicated that the enjoyment was irrelevant as there was consent. | “both consented, just not comfortable” (BU, F) “Enjoyment is irrelevant, as are your motives. You willingly gave consent, definitely not rape.” (ARU, M) |
| Both | 69 (22%) | N/A | 58% of participants highlighted that both people were involved in the decision to consent. | “Consent from both parties” (BU, F)  “We both agreed, full consent, not rape.” (ARU, M) |
| They | 66 (31%) | N/A | In this case, participants used a more inactive tense and put the responsibility more onto the partner or the other person. | "If the situation made the individual uncomfortable, they had every right to stop halfway through" (BU, F)  “they have agreed” (BU, M) |
| Force | 18 (31%) | 4 (50%) | 16% of participants highlighted that these scenarios did not indicate any force, coercion, or persuasion. | “pressured into giving consent and didn’t enjoy” (BU, F)  “doesn't seem to be any pressure or wrong doing on the part of the partner” (ARU, M) |
| Need for communication | 42 (14%) | 0 | Given the bad sex, 37% of participants highlighted how important it is to communicate, with communication determining whether the consent has been withdrawn or not. | "there is no mention of her asking her boyfriend to stop having sex" (BU, F)  “anyone has the right to say no during and end it” (BU, M) |
| Consequences | 0 | 6 (0%) | Participants (5%) indicated that the consequences of bad sex were that it would be stressful. The impact of stress on how the scenario could be perceived was highlighted. | “I imagine the situation being stressful.” (ARU, F) |

| ***Consent – Unwanted – Pleasurable*** | | | | |
| --- | --- | --- | --- | --- |
| ***Category*** | ***Positive Mentions*** | ***Negative Mentions*** | ***Description*** | ***Example of Transcripts*** |
| Consent | 137 (33%) | 43 (43%) | Consent was highlighted as being present and important in these scenarios, however a minority of participants (5%) pointed out that consent may have changed over time given the lack of wanting. | “Partner didnt say no” (BU, F)  “thus giving consent and it is definitely not rape.” (ARU, M) |
| Wanting | 0 | 21 (40%) | 18% of participants seem to acknowledge that these scenarios did not include wanting from both partners, however they end up choosing to engage in the sexual activity. | “didn’t want to, but did agree to” (BU, F)  “I do not want to have sex, but I choose to.” (ARU, M) |
| Pleasure | 14 (36%) | 3 (33%) | In order to justify sex that was not wanted, 12% of participants did acknowledge that the sex was eventually enjoyed and was pleasurable and therefore meant it was not as negative. | “Both people agree to have sex and enjoy it.” (BU, F)  “it is agreed and the people enjoy it” (ARU, M) |
| Both | 18 (18%) | N/A | 16% of participants did indicate that both participants consented. | “both consented although one was hesitant.” (BU, F)  “Not rape as consent is from both parties” (BU, M) |
| They | 91 (31%) | N/A | Participants (66%) were using the second and third person quite frequently when describing the sexual scenarios. | "They are unsure of having sex" (BU, F)  “They agreed to it” (BU, M) |
| Force | 40 (39%) | 7 (25%) | 35% of participants made reference to pressure and insistence from one partner. Depending on how the participant interpreted the event determined their opinions on the matter. | “I think if the person inititated it, but did not pressure me, I would nto consider this rape.” (ARU, F)  “he insisting from the partner is a little pressuring” (ARU, F) |
| Need for communication | 10 (22%) | 5 (0%) | 9% of participants made reference to the fact that the partners should communicate their wants more and a lack of communication indicated a negative relationship. | “If my partner did not want to have sex they shouldn’t have given in and just said no I am not in the mood” (BU, F)  “This is a sign of a bad situation and a bad relationship in general” (ARU, M) |
| Consequences | 2 (0%) | 7 (0%) | Several participants (8%) indicated that the scenarios described here might seem stressful and the negative consequences emotionally might affect how they judged the situation. | “I would feel highly guilty.” (ARU, F) |

| ***Consent – Unwanted – Not pleasurable*** | | | | |
| --- | --- | --- | --- | --- |
| ***Category*** | ***Positive Mentions*** | ***Negative Mentions*** | ***Description*** | ***Example of Transcripts*** |
| Consent | 174 (29%) | 6 (0%) | Participants (45%) highlighted that consent was the key driving factor in these scenarios. While 4% participants did indicate some form of mind changing being important, overwhelming the stated presence of consent was the key factor. | “They agreed” (BU, F)  “What matters is consent. If the partner initiated despite knowing you didn't want to have sex then that was bad form" (ARU, M) |
| Wanting | 2 (50%) | 5 (100%) | Participants were aware that sex could be consensual but unwanted and 6% indicated that the lack of wantedness affected their judgements, but not as many as considered consent important. | “I don't want sex, but I choose to have sex for my partner's sake.” (BU, F)  “They agree and want it in the end” (BU, M) |
| Pleasure | 4 (25%) | 19 (13%) | 17% of participants made reference to the lack of pleasure influencing their decisions. However, 4 mentions referred to the fact that pleasure is irrelevant in how a sexual scenario should be appraised. | “ut because an experience isnt enjoyable that doesn't class it as rape.” (ARU, F)  “behaviour awful during sex” (BU, M) |
| Both | 19 (19%) | N/A | Fewer participants (17%) referred to both parties in these scenarios. | “Both consent” (BU, F) |
| They | 60 (32%) | N/A | Participants (53%) were quite keen to put the responsibility for the bad sexual scenarios onto the characters depicted. | "They didn't want to have sex, however they did agree" (BU, F)  “as they’re consenting” (BU, M) |
| Force | 11 (25%) | 5 (0%) | 9% of participants considered the scenarios depicted coerced sex, whereas 4% indicated that no pressure was used. The participants' appraisal determined how they rated the scenarios. | “doesn’t seem like it felt forced” (BU, F)  “No indication is given that the sex was anything but consensual, or that pressure was applied in anyway.” (ARU, M) |
| Need for communication | 22 (31%) | 0 | Participants (19%) indicated that one partner needed to communicate with the other. The presence (or absence) of such communication would affect how the scenario was appraised. | “body language” (BU, F)  “Definitely not rape. (assuming you didn't tell them to stop half way through, due to their awful behaviour)” (ARU, M) |
| Consequences | 0 | 9 0%) | 8% of participants highlighted how the situation would be quite stressful and this impacted on how they rated the situation. | “Very stressful as your partner dont understand you and you got sacrifice” (ARU, F) |

| ***No Consent – Wanted – Pleasurable*** | | | | |
| --- | --- | --- | --- | --- |
| ***Category*** | ***Positive Mentions*** | ***Negative Mentions*** | ***Description*** | ***Example of Transcripts*** |
| Consent | 57 (34%) | 80 (16%) | Participants focused on whether participants changed their minds (despite no consent being mentioned). The phrase "giving in" meant giving consent to 40% participants but to 30% it did not. | ”It would not be rape as long as he has consented or shown his consent after he has initially refused” (BU, F)  “The phrase "you give in" implies you changed your mind and gave consent. Thus not rape.” (ARU, M) |
| Wanting | 7 (17%) | 12 (22%) | Some participants identified wanting sex (6%), whereas others did not. Much of the commentary here seemed to indicate participants were conflating wanting and consent. | “they both wanted it” (BU, F)  “however I don't want to have sex with my partner" (ARU, M) |
| Pleasure | 48 (17%) | 12 (50%) | While six (5%) participants commented that pleasure is irrelevant, 42% of participants did indicate a mitigating role of pleasure on whether these scenarios could be considered rape. | "is very much borderline. As although they refused multiple times, they enjoyed the act in the end." (BU, F)  “The person has engaged in the activity and found pleasure” (BU, M) |
| Both | 10 (30%) | N/A | 8% of participants referred to both parties in these scenarios. |  |
| They | 45 (26%) | N/A | 39% of participants were quite keen to put the responsibility for the bad sexual scenarios onto the characters depicted. | "It appears that they almost changed their mind" (BU, F)  “As they’ve rejected initiation it means they haven’t given consent” (BU, M) |
| Force | 13 (40%) | 3 (50%) | 11% of participants highlighted that there was some pressure, coercion, and force to ensure that sex occurs. Some participants (5%) indicated that some degree of pressure was acceptable, especially within relationships. | "They did not give full consent, only after being pressured into it" (BU, F)  “forced behaviour” (BU, M) |
| Need for communication | 0 | 1 (0%) | In these scenarios, participants did not consider that there was need to communicate or that communication between the partners would mitigate the appraisal of these situations. | “partner engages and doesn’t say no” (BU, F) |
| Consequences | 7 (0%) | 2 (0%) | Largely, 6% of participants focused on the consequences that were unrelated to whether the sex was consensual or not (focusing on superfluous information). Nonetheless, there were limited consistency on how the stress caused would impact on whether the situation was rape. | “It will be stressful considering the person has been pressured into it” (BU, F)  “backing down on sexual safety csn be dangerous” (BU, M) |

| ***No Consent – Wanted – Not Pleasurable*** | | | | |
| --- | --- | --- | --- | --- |
| ***Category*** | ***Positive Mentions*** | ***Negative Mentions*** | ***Description*** | ***Example of Transcripts*** |
| Consent | 62 (58%) | 117 (38%) | 50% of participants identified that there was a lack of consent in these scenarios and that this was critical for appraising the scenarios. However, there were some comments (6% of participations) regarding that consent was implied or present because of the relationship. | “if it was a partner, and I started to have sex I would automatically feel like I should oblige.” (ARU, F)  “no consent” (BU, M) |
| Wanting | 0 | 12 (50%) | 11% of participants conflated the lack of consent with a lack of wanting, despite the scenarios indicating the sex was wanted. | “hard to have sex with a male if they didnt want to, there shouldn’t be pressure though” (BU, F)  “I do not want to have sex, but I give in to my partner's desire” (ARU, M) |
| Pleasure | 0 | 62 (21%) | Nine (7%) participants highlighted that pleasure is irrelevant when judging whether the scenario depicted rape or not. When it was used, participants highlighted the lack of pleasure as contributing to a description of rape. | “So I would not feel comfortable or respected in this situation and would definitely feel it was not right.” (ARU, F)  “The fact that you didn't enjoy it is irrelevant,” (ARU, M) |
| Both | 12 (67%) | N/A | Participants did not consider both to have been involved in these conditions (9% did). | “both agreed to have sex” (BU, F)  “Although it is morally wrong, both have agreed.” (BU, M) |
| They | 56 (35%) | N/A | Participants (43%) were quite keen to put the responsibility for the bad sexual scenarios onto the characters depicted. | "you refused and they pressed on regardless" (BU, F)  “They refused” (BU, M) |
| Force | 32 (43%) | 5 (20%) | Participants indicated that the presence of force affected their judgements. However, some participants interpreted the scenarios as to not be involving force or pressure. 11% also implied that pressure and insistence is okay if the person changes their mind. | “they may be aroused but if they do not consent then it is forced and therefore rape” (BU, F)  “If they used force, that's a different matter.” (ARU, M) |
| Need for communication | 17 (44%) | 1 (0%) | Participants described these situations as resulting from a bad relationship or where one partner has limited awareness of the other's needs. However, the majority of comments here indicate that the receiver needs to communicate to their partner to stop before it is considered rape. | “It can only be classed as rape if you tell your partner to stop and they don't” (ARU, F)  “this shouldn’t happen in a relationship, and if it does it should be discussed” (BU, M) |
| Consequences | 0 | 5 (50%) | Participants typically separated the consequences from the appraisal, but when they were linked (4%), participants did consider stress to impact on whether the scenario depicted rape or not. | “caused distress” (BU, F)  “it is just stressful , not rape however I may not enjoy it that much” (ARU, M) |

| ***No Consent – Unwanted – Pleasurable*** | | | | |
| --- | --- | --- | --- | --- |
| ***Category*** | ***Positive Mentions*** | ***Negative Mentions*** | ***Description*** | ***Example of Transcripts*** |
| Consent | 31 (29%) | 87 (45%) | Participants (27%) did identify that there was a lack of consent in these scenarios. However, 4% of participants did indicate that pressure and force as part of a relationship negates the need for obvious consent. 9% of participants did indicate that there was evidence of giving in. | “It's a tricky one because i intepret this as being almost a form of seduction and eventually it sounds consentual.” (ARU, M)  “dubious consent but consent nonetheless” (ARU, F) |
| Wanting | 1 (0%) | 13 (60%) | 11% of participants did indicate that the sex was unwanted and this contributed to why they appraised a situation as rape or not. | “They didn’t want to” (BU, F)  “I chose not to have sex, but then give in to my partner's desire to have sex.” (ARU, M) |
| Pleasure | 51 (53%) | 7 (33%) | 45% of participants did indicate that the presence of pleasure changed how the situation was appraised. Pleasure might have led to retrospective consent. Ten participants (8%) did highlight that pleasure is irrelevant in the appraisal of rape. | “pressuring someone to have sex with you but you enjoyed it so 50/50” (BU, F)  “However, if you enjoyed yourself eventually then, presumably, you retrospectively give consent, and agree that you don't mind what the person did.” (ARU, M) |
| Both | 9 (22%) | N/A |  | “both parties did not consent” (BU, F)  “both enjoyed the experience” (BU, M) |
| They | 47 (47%) | N/A | Participants (41%) did distance themselves from these scenarios and put more responsibility onto the receiver. | "they continued and didn't technically refuse" (BU, F)  “they did reject, after that it could be manipulation” (BU, M) |
| Force | 118 (34%) | 2 (0%) | Participants' interpretation of force and pressure affected how they appraised the situation. When participants felt that there was more force, they considered the scenario to represent rape. However, 4% of participants highlighted how pressure may be acceptable within relationships. | “if you enjoy that pressure it may become routine which makes hte lines of consent extremely blurred.” (ARU, F)  “If they are being forceful in the way they persist then I'd be more likely to rate it as rape.” (ARU, M) |
| Need for communication | 5 (100%) | 1 (0%) | There were few mentions indicating that the partners should communicate their refusal to have sex more (3% of participants). | “you have the option to say no its just that you haven't.” (ARU, F)  “it does not state that they changed their mind” (BU, M) |
| Consequences | 0 | 5 (0%) | 4% of participants mentioned that the situation might be stressful, but only in the beginning when the consent was not given. | “initially stressful” (ARU, F) |

| ***No Consent – Unwanted – Not Pleasurable*** | | | | |
| --- | --- | --- | --- | --- |
| ***Category*** | ***Positive Mentions*** | ***Negative Mentions*** | ***Description*** | ***Example of Transcripts*** |
| Consent | 15 (64%) | 110 (31%) | Largely, 32% of participants identified a lack of consent in these scenarios, but many found them harder to empathise with. Participants also qualified the consent, indicating that it was given through persistence or pressure (8%). | “Even though they feel very uncomfortable during sex they havent asked you to stop and they did consent before having sex even if they didnt actually want to” (BU, F)  “there is rejection and them insisting that it happens” (ARU, M) |
| Wanting | 0 | 20 (33%) | 18% of participants identified that the sexual scenarios depicted unwanted sex and this contributed to their appraisals. | “They didn’t want it” (BU, F)  “I do not want to have sex” (ARU, M) |
| Pleasure | 0 | 43 (30%) | 38% of participants used the lack of pleasure to highlight that these situations depict rape. | “and they don’t enjoy it” (BU, F)  “because the pleasure isn't there and the person continues I'm more inclined to rate it as rape.” (ARU, M) |
| Both | 3 (0%) | N/A |  |  |
| They | 66 (33%) | N/A | 58% of participants did distance themselves from these scenarios and put more responsibility onto the receiver. | "they were forced" (BU, F)  “They have been coerced” (BU, M) |
| Force | 52 (44%) | 1 (100%) | Different participants interpreted pressure and force differently. 39% of participants indicated any pressure as leading to rape, whereas others (14%) indicate that some pressure and insistence is acceptable. | “to insist someone is to force” (ARU, F)  “Do we mean they just vocally insisted and you gave in and agreed? If so then not rape. If they forced you in any way, then definitely rape.” (BU, F) |
| Need for communication | 4 (0%) | 2 (0%) |  |  |
| Consequences | 0 | 3 (0%) | 2% of participants that used consequences indicated that the stressful would affect how they interpret the situation. | “I might be stressed but can deal with that situation” (ARU, M) |

Table S2.

*Content Analysis tables for Study 2. Tables include content categories, the number of times the category was mentioned as being used to make judgements about rape, the number of times the lack of the category was used to make judgements about rape (the top value is the subject condition and the bottom value is the initiator condition), an explanation of how the category was used, and example quotes.*

| ***Consent – Wanted – Pleasurable*** | | | | |
| --- | --- | --- | --- | --- |
| ***Category*** | ***Positive Mentions*** | ***Negative Mentions*** | ***Description*** | ***Example of Transcripts*** |
| Consent | 136  134 | 0  0 | Most participants used consent to appraise the situations. | "Consented" |
| Wanting | 2  12 | 0  0 | Few participants highlighted wanting as a reason to appraise the situation. When they did, it appeared to be used as a synonym for consent. | "Both wanted to do it" |
| Pleasure | 26  42 | 0  0 | Pleasure was used by some participants to justify their ratings. This was more evident in the initiator condition than the subject condition. | "both agree to have sex and both enjoy it" |
| Both | 97  123 | N/A | Many participants highlighted that both partners were involved and gave their consent. This highlighted a mutual nature of these sexual scenarios. | "Both consented" |
| They | 1  3 | N/A | Few participants used a less active phrasing than in other conditions. |  |
| Force | 0  0 | 4  1 | Where force was mentioned, it was highlighted that there was an absence of force that influence participants' appraisals. | "both people initiated and there was were no force" |
| Need for communication | 0  0 | 0  0 |  |  |
| Consequences | 2  3 | 0  0 | The few mentions regarding the consequences highlighted that the sex was positive. | "everyone had fun so yay" |

| ***Consent – Wanted – Not Pleasurable*** | | | | |
| --- | --- | --- | --- | --- |
| ***Category*** | ***Positive Mentions*** | ***Negative Mentions*** | ***Description*** | ***Example of Transcripts*** |
| Consent | 89  92 | 3  5 | Most participants thought consent was an important factor in determining whether these scenarios represented rape or not. | "Consent was given" |
| Wanting | 1  7 | 1  5 | A few participants used the presence of wanting to appraise the situations. Notably, more participants used wanting as a key variable when they were the initiators of the encounter than the subjects. | "They still wanted to have sex" |
| Pleasure | 0  0 | 16  19 | The lack of pleasure did contribute to the appraisal process for some participants, with several highlighting that the lack of enjoyment caused more stress and made the scenario seem more like rape. | "They felt uncomfortable during the process" |
| Both | 46  40 | N/A | Many participants referred to both participants in the sexual encounter highlighting the more equal nature of these scenarios. | "Both people agreed" |
| They | 24  37 | N/A | The language used to describe these scenarios was somewhat less active than in the consensual, wanted, and pleasurable scenarios. | "They said yes" |
| Force | 4  5 | 1  5 | There was some disagreement among participants about whether the scenarios had force in them (due to the lack of pleasure). Nonetheless, the interpretation guided how participants appraised the scenarios. | "They are not pressured into it" |
| Need for communication | 50  34 | 0  0 | Imply participant should know others consent - also responsibility on partner to say stop | "if they feel uncomfortable they should know that they can stop sex at any time" |
| Consequences | 0  0 | 10  7 | Several participants highlighted the potential stress of the scenario and this impacted on their judgements. Some participants, however, pointed out that the stress caused by the sex is unrelated to rape. | "aftermath isn't anything to do with rape" |

| ***Consent – Unwanted – Pleasurable*** | | | | |
| --- | --- | --- | --- | --- |
| ***Category*** | ***Positive Mentions*** | ***Negative Mentions*** | ***Description*** | ***Example of Transcripts*** |
| Consent | 84  101 | 6  11 | Consent was used by many participants to judge the situations. Some participants highlighted the changing nature of consent depicted (thereby conflating wanting and consent). Other people suggested that the characters let the sex happen. | "but consented in the end" |
| Wanting | 0  5 | 26  28 | Participants did use wanting to determine if the scenarios depicted rape or not. Typically, they highlighted the lack of wanting impacted on their judgements of rape. | "Initially did not want to do it" |
| Pleasure | 8  24 | 0  7 | Participants did use the presence of pleasure to help appraise the situation. The presence of pleasure negated the lack of initial wanting. This was especially noticeable in the initiator conditions. | "and if they started enjoying it then consent is surely there" |
| Both | 12  16 | N/A | For these scenarios, participants were less likely to describe them as involving both participants involved in the decision. | "Both consented" |
| They | 26  90 | N/A | Especially for the initiator conditions, participants were more likely to put the responsibility onto the other person but using the pronoun "they." | "They did not want to" |
| Force | 23  15 | 3  7 | Generally, participants used the implied presence of force to influence their judgements. With increased perceptions of force or pressure, participants were more likely to consider the scenario rape. | "I wouldn't consider this rape unless you forced them" |
| Need for communication | 5  15 | 0  0 | Especially in the initiator condition, participants highlighted that the subject should communicate better with their partner. In this way, there was some indication of shifting the blame onto the receiver. | "If they did not want to have sex with their partner, they should be able to tell them" |
| Consequences | 0  1 | 9  6 | Participants did indicate that the scenario might be stressful and this would impact on how they appraised the situation. | "but still very stressful and upsetting" |

| ***Consent – Unwanted – Not pleasurable*** | | | | |
| --- | --- | --- | --- | --- |
| ***Category*** | ***Positive Mentions*** | ***Negative Mentions*** | ***Description*** | ***Example of Transcripts*** |
| Consent | 85  103 | 5  8 | The use of consent to determine whether the situation is rape was clear, however there was a tendency for participants to describe the consent in less confident terms. | "They did consent" |
| Wanting | 0  1 | 18  34 | Many participants highlighted that the lack of wanting did contribute to their appraisals of the situation. | "They don't want sex" |
| Pleasure | 0  0 | 17  28 | While several participants did use the lack of pleasure to guide their appraisals, there were a few who highlighted that the presence of pleasure was irrelevant in such an appraisal. | "Gave consent, doesn't matter about how good the sex is." |
| Both | 3  6 | N/A | Few participants used the word "both" in their descriptions, highlighting that the scenarios were more one sided. | "both have agreed to it" |
| They | 25  91 | N/A | Participants used a less active phrasing when describing their appraisals of the situation. These were more frequent in the initiator condition. | "They agreed" |
| Force | 7  21 | 9  8 | Several participants highlighted the presence or absence of pressure and force in these scenarios. There was disagreement among participants, and participants highlighted that different levels of force/pressure would affect how they appraised the situation. | "your partner was implicitly pressured into consenting. You did not expressly pressure them..." |
| Need for communication | 23  19 | 0  0 | Several participants highlighted that communication was vital for appraising the situation. Participants suggested that they would appraise the situation as rape more if the character did not say they were not enjoying it. This highlights further that several participants blame the subject for the encounter. | "Although they did not want to have sex, they did not say this and agreed to sex." |
| Consequences | 0  0 | 9  4 | For these scenarios, the consequences that influenced participants' appraisals were more to do with how regret the character had after the event. | "they do regret it" |

| ***No Consent – Wanted – Pleasurable*** | | | | |
| --- | --- | --- | --- | --- |
| ***Category*** | ***Positive Mentions*** | ***Negative Mentions*** | ***Description*** | ***Example of Transcripts*** |
| Consent | 30  24 | 44  49 | Participants did make reference to the fact that sex was not consented in these scenarios and used this to make their judgements. However, there was some disagreement about whether consent was present or not among the participants. | "Rejection and pressured, so light rape, however enjoyment so eventually it was wanted instead of unwanted" |
| Wanting | 4  7 | 11  13 | Participants did use wanting in their judgements of rape, with some participants conflating wanting and consent in their appraisals. | "if the sex is still wanted it is not rape" |
| Pleasure | 25  25 | 3  5 | Pleasure was used to mitigate the judgements of rape by several participants. Because the situations were pleasurable, participants felt that the scenarios did not directly represent rape. | "they still pushed you into it which is a bit wrong, but its okay as you liked it." |
| Both | 7  5 | N/A | Only a few participants made reference to both partners in these scenarios, and these references were made when the scenario was judged as consensual. | "both consented and found it enjoyable" |
| They | 25  47 | N/A | Many participants, especially in the initiator condition made use of the third person tense in their descriptions. This represents some form of psychological distancing. | "They rejected and had to be pressured into it" |
| Force | 58  70 | 4  0 | Many participants referred to the presence of force or persuasion in these scenarios. The appraisal and interpretation of the amount of force used was used to legitimise the participants ratings. | “Fine line between pressure and continual flirting.” |
| Need for communication | 3  2 | 0  0 | Where communication was used to determine participants rating, it highlighted that the subject should communicate their refusal to have sex more. | "it is their responsibility to refuse" |
| Consequences | 0  3 | 7  0 | The presence of stress impacted on how participants appraised these situations, with participants thinking that they were less like rape if no distress occurred, even if the scenario did not depict consensual sex. | "but no distress occured" |

| ***No Consent – Wanted – Not Pleasurable*** | | | | |
| --- | --- | --- | --- | --- |
| ***Category*** | ***Positive Mentions*** | ***Negative Mentions*** | ***Description*** | ***Example of Transcripts*** |
| Consent | 10  1 | 83  104 | The majority of participants identified and used the fact that these scenarios did not depict consent in their appraisals of rape. | "no consent" |
| Wanting | 0  3 | 12  23 | Participants also used wanting in their appraisals of rape, however, the grammatical structures indicated that participants were conflating wanting and consent when making such judgements. | "They don't want to" |
| Pleasure | 0  0 | 13  36 | The lack of pleasure was used by participants when making their judgements of rape. This was especially noticeable in the initiator condition. | "This is rape because your partner does not consent and does not enjoy it" |
| Both | 1  0 | N/A |  |  |
| They | 27  69 | N/A | More noticeable in the initiator condition, the psychological distancing in language use with the word "they" was obvious. Participants indicated that the partner, the subject had more responsibility for the scenario than in more consensual and pleasurable scenarios. | "They might have agreed at the start, but that can change throughout" |
| Force | 35  36 | 1  3 | The presence of force or pressure did impact on participants judgements of rape. Some felt that any presence of force meant that the scenario depicted rape, whereas others felt that force and pressure was acceptable. | "its not rape but it is unfair to force sex upon your partner" |
| Need for communication | 3  5 | 0  0 | Communication (or lack thereof) was used by participants when making their judgements. Participants felt that the subject should communicate their lack of pleasure and their non consent more vehemently. | "They should state as soon as they are uncomfortable" |
| Consequences | 0  0 | 13  3 | The negative consequences did impact on some participants judgements of the scenarios. The stress experienced by the subject led to some participants considered the scenarios depicted rape. | "seems stressful" |

| ***No Consent – Unwanted – Pleasurable*** | | | | |
| --- | --- | --- | --- | --- |
| ***Category*** | ***Positive Mentions*** | ***Negative Mentions*** | ***Description*** | ***Example of Transcripts*** |
| Consent | 10  12 | 40  62 | Participants used the lack of explicit consent to inform their judgements about these scenarios. | "Enjoyment, however no consent therefore not full rape but form of rape as no consent." |
| Wanting | 1  0 | 8  15 | The lack of wanting sex impacted on how these scenarios were appraised. Lack of wanting did lead to the scenarios being rated as rape. | "They did not initially want to have sex" |
| Pleasure | 11  31 | 8  17 | While there was some disagreement about whether these scenarios were pleasurable, with some participants taking offence to the idea that non-consensual scenarios could be pleasurable, pleasure was used to appraise the situations. Where the situation was pleasurable, participants used this to mitigate the lack of consent, with some participants thinking that pleasure meant it was okay not to have consent. | "enjoyment was gained therefore not full rape."  "but luckily they enjoyed it" |
| Both | 0  1 | N/A |  |  |
| They | 19  60 | N/A | Especially in the initiator condition, participants used psychological distancing in their appraisals of the scenarios. | "they refused sex" |
| Force | 46  76 | 0  0 | Participants made reference to the presence of pressure and force in their appraisals. Such presence made them feel the scenarios depicted rape. | "They were pressured." |
| Need for communication | 0  1 | 0  0 |  | "perhaps that sound be discussed" |
| Consequences | 0  3 | 3  3 | A few participants referred to the stress of the situation and how this impacted on whether the situation depicted rape or not. | "they said no to begin with so quite distressing" |

| ***No Consent – Unwanted – Not Pleasurable*** | | | | |
| --- | --- | --- | --- | --- |
| ***Category*** | ***Positive Mentions*** | ***Negative Mentions*** | ***Description*** | ***Example of Transcripts*** |
| Consent | 13  7 | 45  79 | Consent was used by the majority of participants to appraise the situation. For most, it was relatively clear cut, but some participants indicated that consent was offered after giving in and this meant there was consent. | "They shouldn't have given in" |
| Wanting | 0  0 | 7  35 | The lack of wanting did affect how participants appraised these scenarios, with a lack of wanting leading to more opinions of rape. | "they didn't want it" |
| Pleasure | 0  0 | 13  32 | The lack of enjoyment and pleasure was also used by participants in their appraisals of the situation. | "did not enjoy" |
| Both | 0  0 | N/A |  |  |
| They | 21  91 | N/A | Many participants psychological distanced themselves by putting the responsibility onto the partner, especially in the initiator condition. | "they gave consent but it seemed pressured" |
| Force | 42  91 | 0  2 | The use of pressure and force was used by many participants when they were considering whether the scenario depicted rape. When force was obvious, participants considered the scenario to represent rape. | "They pressurised the person" |
| Need for communication | 0  0 | 0  1 | Only one person mentioned that communication should be maintained, with continuous refusal required otherwise it's not really rape. | "They refused but then gave in and do not continue to refuse, so it's not really rape." |
| Consequences | 0  0 | 6  5 | A few participants felt that the negative psychological consequences would impact on how the situation would be appraised. | "I would regret it" |
